# Genome Analysis of Japanese *Yersinia pseudotuberculosis* Strains Isolated From Kawasaki Disease Patients and Other Sources and Their Phylogenetic Positions in the Global *Y. pseudotuberculosis* Population

**DOI:** 10.1111/1348-0421.13199

**Published:** 2025-01-09

**Authors:** Kazuaki Yasuoka, Yasuhiro Gotoh, Itsuki Taniguchi, Debora Satie Nagano, Keiji Nakamura, Yumi Mizuno, Tomoko Abe, Yoshitoshi Ogura, Hiroshi Nakajima, Masayoshi Uesugi, Masaru Miura, Kazuko Seto, Yuki Wakabayashi, Junko Isobe, Takashi Watari, Sonoko Senda, Noboru Hayakawa, Eiki Ogawa, Toshio Sato, Etsuro Nanishi, Yasunari Sakai, Atsushi Kato, Ippei Miyata, Kazunobu Ouchi, Shouichi Ohga, Toshiro Hara, Tetsuya Hayashi

**Affiliations:** ^1^ Department of Bacteriology, Graduate School of Medical Sciences Kyushu University Fukuoka Japan; ^2^ Department of Pediatrics, Graduate School of Medical Sciences Kyushu University Fukuoka Japan; ^3^ Advanced Genomics Center, National Institute of Genetics Shizuoka Japan; ^4^ Division of Microbiology, Department of Infectious Medicine Kurume University School of Medicine Fukuoka Japan; ^5^ Kawasaki Disease Center, Fukuoka Children's Hospital Fukuoka Japan; ^6^ Okayama Prefectural Research Center of Environment and Public Health Japan; ^7^ Department of Cardiology Tokyo Metropolitan Children's Medical Center Tokyo Japan; ^8^ Osaka Institute of Public Health Osaka Japan; ^9^ Toyama Institute of Health Toyama Japan; ^10^ General Medicine Center, Shimane University Hospital Shimane Japan; ^11^ Integrated Clinical Education Center, Kyoto University Hospital Kyoto Japan; ^12^ Hyogo Prefectural Kobe Children's Hospital Hyogo Japan; ^13^ Department of General Pediatrics Aichi Children's Health and Medical Center Aichi Japan; ^14^ Japan Microbiological Laboratory Miyagi Japan; ^15^ Kawasaki Medical School Okayama Japan; ^16^ Reiwa Health Sciences University Fukuoka Japan

**Keywords:** Japanese strains, Kawasaki disease, phylogenomic analysis, *Yersinia pseudotuberculosis*

## Abstract

*Yersinia pseudotuberculosis* (Ypt) is a gram‐negative bacterium that infects both humans and animals primarily through fecal‒oral transmission. While Ypt causes acute gastroenteritis in humans, an association with Kawasaki disease (KD), a disease that primarily affects infants and young children and causes multisystemic vasculitis, has also been suspected. Although KD represents a significant health concern worldwide, the highest annual incidence rate is reported in Japan. Previously, a geographical origin‐dependent population structure of Ypt comprising the Asian, transitional, and European clades was proposed. However, genomic data on KD‐associated Ypt strains is currently unavailable. In this study, to analyze the phylogenetic and genomic features of KD‐associated strains, we determined the whole‐genome sequences of 35 Japanese Ypt strains, including 11 KD‐associated strains, and constructed a genome set (*n* = 204) representing the global population of Ypt by adding publicly available Ypt genomes. In a phylogenetic analysis, all sequenced Japanese strains, including the KD‐associated strains, belonged to the Asian clade, which appeared to be the ancestral clade of Ypt, and the KD‐associated strains belonged to multiple lineages in this clade. Strains from patients with Far East scarlet‐like fever (FESLF), a KD‐related disease, also belonged to the Asian clade. Moreover, no KD strain‐specific genes were identified in pan‐genome‐wide association study analyses. Notably, however, the gene encoding a superantigen called *Yersinia pseudotuberculosis*‐derived mitogen (YPM) showed a distribution pattern highly biased to the Asian clade. Although further studies are needed, our results suggest that Asian clade strains may have a greater potential to trigger KD.

**Abbreviations**
Cif: cycle‐inhibiting factor
FESLF: Far East scarlet‐like fever
KD: Kawasaki disease
ONT: Oxford Nanopore Technologies
SNP: single‐nucleotide polymorphism
WGS: whole‐genome sequence
YPM: *Yersinia pseudotuberculosis*‐derived mitogen
Ypt: *Yersinia pseudotuberculosis*

## 1 Introduction

*Yersinia pseudotuberculosis* (Ypt) is a facultatively anaerobic gram‐negative bacterium that infects both humans and animals primarily through fecal‒oral transmission. While it usually causes acute gastroenteritis in humans, the infection can progress to septicemia, which significantly increases the risk of mortality [1, 2, 3]. In addition, Ypt has been suspected to be associated with Kawasaki disease (KD), a disease that primarily affects infants and young children and provokes multisystemic vasculitis [4], as patients with Ypt infection who fulfilled the clinical criteria of KD have been reported [5, 6, 7]. A recent prospective study by Hayashi et al. further explored this association in a cohort of KD patients [8]. In cases where Ypt infection coexists with KD (Ypt‐KD), not only gastrointestinal symptoms but also major clinical symptoms of KD and a high incidence of coronary artery aneurysms are often observed. The major symptoms of KD are as follows: (1) fever lasting more than 5 days; (2) bilateral conjunctival injection without exudate; (3) red, dry, cracked lips and a red, swollen tongue; (4) polymorphous rash; (5) cervical lymphadenopathy (greater than 1.5 cm in diameter); and (6) changes in the extremities (including erythema of the palms and soles, edema of the hands and feet, and periungual desquamation). This association suggests that Ypt could be a contributing factor in the pathogenesis of KD, particularly in patients who present with severe coronary complications [5, 6, 7]. Although KD represents a significant global health concern [6, 9], the highest annual incidence rate is reported in Japan [10]. Recent studies have reported an incidence rate of 370 cases per 100,000 children aged 0–5 years in Japan [10, 11]. Despite extensive research, the specific etiology and triggers of KD remain elusive, with the current theory suggesting a complex interplay of genetic predisposition and environmental factors. Epidemiological studies have consistently shown a strong correlation between KD and Asian ethnicity. For example, the incidence of KD among Japanese Americans in Hawaii was notably greater than that among Caucasian children, suggesting that genetic factors may play a significant role in disease pathogenesis [12]. Several environmental and infectious triggers have been suspected, among which Ypt has emerged as a bacterium of interest, especially in the context of Japanese KD patients [6, 7]. Interestingly, a previous phylogenomic analysis of Ypt revealed the geographical origin‐dependent population structure of this species, and Asian, transitional, and European clades were proposed. Japanese isolates were found in the Asian clade along with strains from other regions in Asia [13]. The Japanese strains analyzed in that study were of human origin. However, as the details of the isolation sources of these Japanese strains are unknown, genomic and phylogenetic information on KD‐associated Ypt strains are currently not available.

Ypt infections have also been associated with Far East scarlet‐like fever (FESLF), a distinct clinical manifestation with systemic inflammatory symptoms [14, 15]. Patients with FESLF often exhibit symptoms that overlap with KD, such as fever and rash. FESLF, first reported in Russia, is now recognized as a unique manifestation of Ypt infection that may help to further understand the disease mechanisms potentially linked to KD [6]. Ypt strains associated with FESLF have been characterized by several unique features, such as a specific plasmid profile (frequent possession of the pVM82 plasmid in addition to the pYV virulence plasmid carried by most Ypt strains) [14, 15, 16, 17]. Genome sequences of two strains isolated from FESLF patients are also available in public databases. However, the phylogenetic relationship between KD‐ and FESLF‐associated Ypt strains has not yet been analyzed.

In this study, we determined the whole‐genome sequences (WGSs) of Japanese Ypt strains derived from KD patients and from various other sources, including patients with gastroenteritis, nonhuman animals, and the environment. Using the WGS data of these Japanese strains and those of strains from other geographic regions, which are available in public databases, we performed a large genomic analysis of Ypt to determine the phylogenomic positions of KD‐associated strains in the near‐global population structure of Ypt and to identify any genomic features unique to KD‐associated strains.

## 2 Materials and Methods

### 2.1 Genome Sequencing

The Japanese Ypt strains sequenced in this study (*n* = 35) are listed in Table S1. The strains were cultured overnight at 37°C in lysogeny broth (LB) under aerobic conditions with shaking, and their genomic DNA was extracted and purified using the DNeasy Blood & Tissue Kit (Qiagen). Libraries for Illumina sequencing were prepared using the QIAseq FX DNA Library Kit (Qiagen) and sequenced using Illumina MiSeq to generate paired‐end reads (301 bp ×2). Illumina reads were filtered and trimmed using the Platanus_trim tool with default settings and assembled using Platanus_B v1.1.0 [18]. Sequence quality was evaluated using CheckM v1.1.10 [19]. The completeness and contamination of the draft sequences obtained were greater than 99% (completeness) and lower than 1% (contamination).

Among the 35 strains, seven were subjected to long‐read sequencing using MinION with R9.4.1 flow cells (Oxford Nanopore Technologies [ONT]) to obtain finished genomes. Libraries were prepared using the Rapid Barcoding Kit (ONT). Initially, we attempted to assemble all seven strains using the same pipeline; ONT reads were trimmed and filtered using Porechop v0.2.2 with a minimum length of ≥ 2 kb and a quality score of ≥ 10, and these were then assembled with the Illumina reads for each strain using Unicycler v0.4.6 [20]. Because circular chromosome sequences were not obtained for two strains (okayama_3 and okayama_9524), the ONT reads from these two strains were trimmed and filtered using NanoFilt v2.7.1 [21], and only reads with a quality score of at least 10 and a length of 2000 nucleotides or more were retained (‐q 10 ‐l 2000 ‐‐headcrop 100). These filtered and trimmed reads were assembled with the Illumina reads of each strain using Unicycler v0.4.8 [20].

The sequencing statistics of the 35 strains are shown in Table S1. The sequences of the Illumina and ONT reads and the finished sequences obtained in this study have been deposited in DDBJ/EMBL/GenBank under the BioProject accession numbers starting from PRJDB17094 (see Table S1 for the accession numbers of each strain). These strains, with the exception of one strain (strain 4750), are available from the Pathogenic Bacteria Program of the National Bioresource Project (NBRP) in Japan (https://pathogenic-bacteria.nbrp.jp/). Strain 4750 was not deposited in the NBRP due to the lack of permission from the isolating institution.

### 2.2 Sequences Obtained From the NCBI Database

Publicly available genome sequences of Ypt strains were retrieved from the NCBI SRA database (accessed on November 6th, 2023). We first selected strains for which country information and Illumina short reads were available (*n* = 300) and assembled their read sequences using Platanus_B v1.1.0 [18] to obtain draft genomes. After a quality check using CheckM with the criteria of > 99% completeness and < 1% contamination, 48 genomes were excluded, resulting in 252 high‐quality draft genomes. In addition, 13 finished Ypt genomes with country information were obtained from the NCBI database. Thus, 265 publicly available Ypt genomes were used in this study. The draft genome sequence of the type strain of *Yersinia similis* (strain Y228, accession no. GCF_001053095.1) [22] was also retrieved from the NCBI database and used as an outgroup in phylogenetic analyses.

### 2.3 Gene Annotation, Identification of Core Genes, and Phylogenetic Analyses

Gene prediction and annotation were conducted using the DFAST v1.2.0 program [23]. The core genes were identified using the Panaroo v1.2.3 pipeline [24] with a threshold set at 95%, and the core gene single nucleotide polymorphisms (SNPs) were identified using snp‐sites v2.3.2 [25]. For phylogenomic analysis, strain groups with identical core gene SNPs (10 groups, all from the 252 draft genomes from the NCBI database) were identified using Molecular Evolutionary Genetics Analysis X (MEGA X) v10.1.8 [26]. From each group, one strain was randomly selected, resulting in a final set of 204 Ypt genomes (184 draft and 20 finished genomes; see Table S2 for the list of the 169 genome sequences from the NCBI database included in the final set). A maximum likelihood (ML) tree of the 204 Ypt strains was constructed using 65,835 SNPs identified in 3,299 core genes using RAxML v8.2.10 [27]. with the general time reversible (GTR)‐GAMMA model of nucleotide substitution and 500 bootstraps. To determine the root of the tree, an additional phylogenetic analysis including the type strain of *Y. similis* was also performed using SNPs in 3236 core genes. The ML tree of the 204 Ypt strains was generated using iTOL v6 [28]. The summary of strain information used in iTOL is available on the Microbiology Society's Figshare (DOI: 10.6084/m9. figshare.21514572).

### 2.4 Detection of Major Virulence‐Related Genes

The presence/absence of major virulence‐related genes of Ypt in each genome was determined using BLASTX v2.4.0 with a threshold of > 90% sequence identity and > 90% query sequence coverage (see Table S3 for the list of genes examined and the accession numbers of the query sequences). Structural comparisons of the genomic regions containing the *ypmA* or *ypmC* gene were conducted and displayed using GenomeMatcher v3.06 [29].

### 2.5 Pangenome‐Wide‐Association Study (Pan‐GWAS)

A pan‐GWAS analysis was conducted for the entire Ypt strain set (204 strains), with a focus on the comparison between the KD‐associated (*n* = 11) and non‐KD‐associated (*n* = 193) strains. An additional pan‐GWAS analysis of the strains within the Asian clade (69 strains) was also conducted to compare the KD‐associated (*n* = 11) and non‐KD‐associated (*n* = 58) strains. These analyses were performed using Scoary v1.6.16 [30] with default parameters. We selected genes with *p* < 0.05 in the pairwise comparison test, which was designed in Scoary to account for population structure by incorporating phylogenetic relationships and removing the bias from clonally related isolates, and the presence or absence of these genes was manually reexamined.

## 3 Results and Discussion

### 3.1 Genome Sequence Set Analyzed in This Study

We sequenced 35 Japanese Ypt strains in this study. These strains included 11 strains from KD patients and 24 strains from other sources, such as patients with gastroenteritis, nonhuman animals, and water samples (Table 1; see Table S1 for the details of each strain). Among these, seven genomes were completed by hybrid assembly using Illumina short reads and ONT long reads. The number of scaffolds ranged from a minimum of 1 to a maximum of 274, with an average of approximately 119.2. The depth of sequence coverage was greater than 30× for all the genomes.

**Table 1:** Sources of Ypt strains analyzed in this study.

Source of sequence data	Isolation		Sequence status	
	Region (country)	Source	Draft	Finished
This study	Japan	KD	6	5
		non‐KD		
		Human (enteritis)	13	
		Animal	7	2
		Environment	2	
NCBI	Japan	Human	4	1
		Animal	11	
		Environment	2	
		na	1	
	Asia w/o Japan*	Human	2	
	(China, South Korea)	Animal	3	
	Russia	Human	3	
		Human (FESLF)	1	1
		Animal	6	
	Finland	Human	8	
		Animal	11	
		Environment	4	
	Europe w/o Finland**	Human	22	5
		Animal	13	3
		na	6	
	Oceania (New Zealand)	Human	39	1
		Animal	6	
	North America***	Human	2	1
		Animal		1
		na	11	
	South America (Brazil)	Animal	1	
Total			184	20

Abbreviations: FESLF, Far East scarlet‐like fever; KD, Kawasaki disease; na; not available; w/o; without.

* South Korea (1), China (4).

** Sweden (5), Denmark (3), Belgium (4), Germany (4), France (10), Italy (7), United Kingdom (16); the numbers of strains are indicated in parenthesis.

*** USA (13), Canada (2).

In addition, we obtained high‐quality genome sequences of 169 Ypt strains isolated from various geographic regions from the NCBI database, which included 19 Japanese strains (Table S2). Thus, in this study, we analyzed the genomes of 204 Ypt strains. Twenty genomes were finished genomes, and the sizes of the closed chromosomes ranged from 4,367,018 to 5,026,929 bp.

As summarized in Table 1, 114 of the 204 strains were of human origin, 64 were from nonhuman animals, and eight were from environmental samples. Notably, two strains were isolated from patients with FESLF in Russia [16]. The 204 strains were isolated in 16 countries across the world except for Africa; thus, this set can represent the near‐global population of Ypt.

### 3.2 Phylogenetic Positions of KD‐Associated Strains in the Near‐Global Population Structure of Ypt

**Figure 1:**
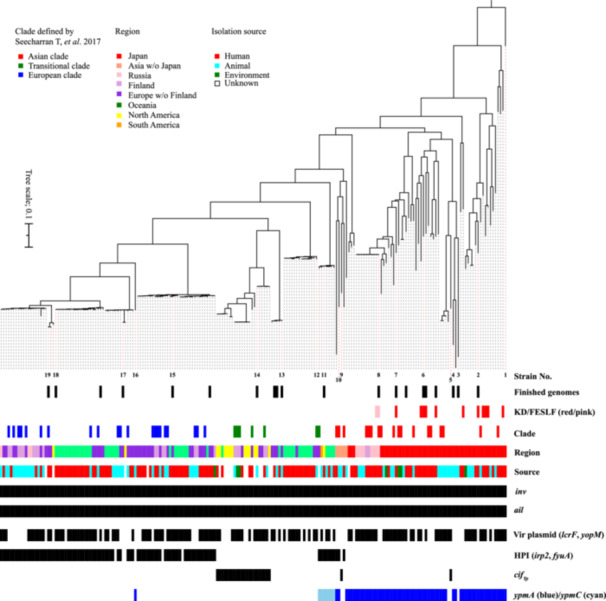
Phylogenetic tree of 204 Ypt strains analyzed in this study. An ML tree was constructed using 65,835 SNP sites on 3299 core genes and rooted by the type strain of *Y. similis*. Below the tree, meta‐information for each strain, along with the presence or absence of genes encoding major virulence factors, is displayed. Nineteen strains (numbered from 1 to 19) were used for the analysis of *ypm* gene‐containing regions (numbers correspond to those in Figure 2). The clades of the strains indicated were defined by Seecharran et al. in 2017 [13]. The presence of the *Yersinia* virulence plasmid was determined by the presence of two marker genes, *lcrF* and *yopM*. The presence of the high pathogenicity island (HPI) was determined by the presence of two marker genes, *irp2* and *fyuA*.

We performed a phylogenetic analysis of the 204 Ypt strains based on their core gene SNPs with the type strain of *Y. similis*, the *Yersinia* species most closely related to Ypt [31], as an outgroup (Figure 1). The results of this analysis confirmed the region‐dependent phylogenetic relationship between Ypt strains [17] and revealed that all Japanese strains belonged to the Asian clade, which separated from *Y. similis* first [22, 31]. Importantly, the 11 KD‐associated strains were dispersed within the Asian clade. This finding indicates that the KD‐associated strains do not belong to any specific subclades/sublineages and thus do not share a specific phylogenetic background.

Note that in this analysis, we did not use *Y. wautersii*, which was previously reported to be most closely related to Ypt [32], as an outgroup. This was because in our preliminary phylogenetic analysis including *Y. wautersii* strains, *Y. wautersii* strains represented a sublineage of the Ypt Asian clade in contrast to the previous report by Savin et al. [33], which showed *Y. wautersii* branches between *Y. similis* and the Ypt Asian clade. A potential reason for this discrepancy is that while we analyzed only Ypt and its close relatives (*Y. wautersii* and *Y. similis*) using their 3299 core genes, Savin et al. analyzed diverse strains covering the entire genus *Yersinia* based on 500 core genes of these strains [33].

### 3.3 Search for KD‐Specific Genes

The 11 KD‐associated strains presented different phylogenetic backgrounds, as described above, but they may contain specific genes that are rarely present in non‐KD‐associated strains. Therefore, using Scoary, we conducted a pan‐GWAS analysis of all 204 strains to search for genes that were enriched in the KD‐associated strains. Although this analysis initially detected 16 genes encoding hypothetical proteins, manual inspection of the presence or absence of each of these genes revealed that, for all 16 genes, an identical gene was present in many non‐KD‐associated strains (because these genes were located at different genomic loci, they were treated as a different gene by the Panaroo pipeline). Therefore, no genes that were significantly enriched in the KD‐associated strains were found. An additional pan‐GWAS analysis was performed on the 69 strains belonging to the Asian clade. This analysis also initially detected 10 genes, but the result obtained by manual inspection of these genes was the same as that obtained for the 16 genes detected in the analysis of all 204 strains. These results suggest that no specific Ypt genes are associated with KD and that if there are any bacterial factors that are linked to (or trigger) the onset of KD, these factors may be common to some specific lineage, such as the Asian clade or a subset of it. Interestingly, the FESLF strains also belonged to the Asian clade (Figure 1); there are notable similarities between KD and FESLF symptoms, such as persistent fever, rash, conjunctivitis, and mucosal inflammation [34], as well as some differences, such as the age of onset, the presence of coronary artery aneurysms, activated T cells, and the response to intravenous immunoglobulin therapy [14].

### 3.4 Specific Distribution of the Genes Encoding the YPM Superantigen in the Asian Clade and Close Relatives in the Transitional Clade

Given that a genome set representing the near‐global population structure of Ypt is available, we investigated the distribution of genes encoding major virulence factors of Ypt in our genome set (Figure 1). These genes included *inv, ail*, *lcrF, yopM, irp2, fyuA, cif*_*Yp*_, *ypmA*, and *ypmC*, of which *lcrF* and *yopM* were selected as marker genes of the virulence plasmid and *irp2* and *fyuA* were selected as marker genes of the high pathogenicity island (HPI) [35, 36, 37, 38, 39, 40, 41]. Among the genes examined, *ail* and *inv*, both encoding factors involved in invasion by Ypt into host cells and other interactions with hosts, were conserved in all the strains examined, which is consistent with the findings of a previous report [40]. The *lcrF* and *yopM* genes were widely distributed among the Ypt strains, but they were not found in many strains (56/204). In these strains, the virulence plasmid was most likely lost during cultivation in laboratories, because the virulence plasmid is prone to being lost during cultivation at 37°C [42]. In contrast, the *irp2* and *fyuA* genes involved in the yersiniabactin‐mediated iron uptake system were present in most European clade strains, as previously reported [43, 44]. However, these genes were also present in a lineage of the transitional clade and a lineage of the Asian clade, the latter of which was phylogenetically most closely related to the transitional clade. This distribution pattern suggests that HPI was acquired by an Asian clade lineage, from which the transitional and European clades emerged, and was deleted from most lineages in the transitional clade. Note that the HPI‐positive Asian strains were isolated in China and South Korea. It is known that the HPI was horizontally acquired [45] and can be excised by the combined actions of its cognate integrase and a recombination directionality factor Hef [46]. Conversely, the *cif‐like* gene (*cif*_*Yp*_) encoding a homolog of the cycle‐inhibiting factor (Cif), which was first identified as a type III secretion system effector in enteropathogenic and enterohemorrhagic *Escherichia coli* strains [47] and belongs to a family of bacterial toxins called cyclomodulins that modulate the host cell cycle, was present in one lineage in the transitional clade and sporadically in the Asian clade (two phylogenetically unrelated strains). The Cif‐like protein of Ypt (Cif_Yp_) is a functional homolog of Cif in *E. coli* [35].

Interestingly, the *ypmA* gene encoding the YPMa superantigen [48, 49, 50] was present in most Asian clade strains, including not only KD‐associated strains but also FESLF strains isolated in Russia (Figure 1). One European clade strain (YER_AA2585AA; a horse‐derived strain isolated in Denmark) contained the *ypmA* gene. The YPMa protein in this strain had an identical amino acid sequence to those in Asian clade strains and shared the same chromosomal location as described below. The *ypmC* gene encoding YPMc [51], a variant of YPMa containing a single amino acid substitution, was present in the transitional clade but only in the lineage most closely related to the Asian clade. This distribution pattern of YPM (highly biased to the Asian clade and highly conserved within the clade) may be linked to the high incidence rate of KD in Japan [12]; for example, YPM‐producing strains may have greater potential to trigger the onset of KD and its related disease, FESLF, as previously suspected [14, 52, 53]. FESLF has been associated with Ypt strains carrying the *ypm* gene, where the YPM superantigen induces the proliferation of activated T cells (atypical lymphocytes). These strains carry the pVM82 plasmid and the genes on this plasmid may also contribute to their pathogenicity [14, 15, 16, 17]. However, increase in atypical lymphocytes (activated T cells) was not observed in patients with KD associated with Ypt in previous studies including our unpublished data [9, 34]. In addition, while there has been no reports to document coronary artery lesions (CALs) in FESLF patients [14], CALs are often observed in KD patients. Moreover, KD patients infected with Ypt are more likely to develop CALs than KD patients without this pathogen [9]. Notably, cases of KD associated with *Yersinia enterocolitica*, which lacks the *ypm* gene, have also been reported [54]. These findings suggest that while the *ypm* gene may play a crucial role in the pathogenesis of FESLF, its involvement in KD may be supportive [14, 55]. It may be possible that pyroptosis triggered by *Yersinia* infection plays a more significant role in the development of KD [54, 56].

### 3.5 Localization of the *ypm* Gene at the Replication Terminus and Highly Variable Structures of This Region Between Ypt Strains

**Figure 2:**
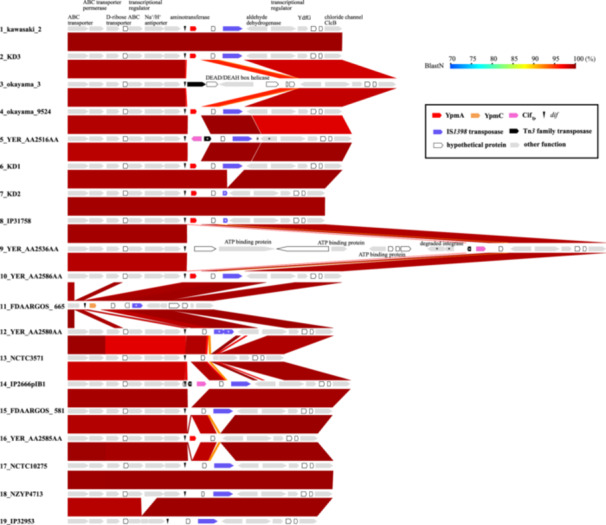
Variation in the genetic organization of the *ypm*‐containing chromosome region. The *ypm*‐containing chromosome regions of nine *ypm*‐positive Ypt genomes and the corresponding chromosome regions of ten *ypm*‐negative Ypt genomes were compared. These 19 Ypt genomes were selected to cover the entire Ypt population. *indicate apparently degraded or remnant protein‐coding genes.

Previously, the *ypm* gene was reported to be located in an unstable chromosome region containing a sequence homologous to the *dif* sequence, the target of Xer site‐specific tyrosine recombinase that resolves chromosome dimers at the replication terminus [57] and that IS*1398* is colocalized in this region [58]. To better understand the relationship between the highly biased distribution of *ypm* and the instability of the *ypm*‐containing chromosome region, we analyzed the genetic organizations of *ypm*‐containing regions as well as corresponding chromosome regions in *ypm*‐negative strains using the sequences of 19 Ypt genomes. These genomes were selected to cover the entire Ypt population (see Figure 1 for their phylogenetic positions). As shown in Figure 2, this analysis revealed that all *ypm* genes, including *ypmC*, were located next to the *dif* sequence in the region corresponding to the replication terminus of the Ypt chromosome. This region, particularly the region from the *dif* locus to the gene encoding an aldehyde dehydrogenase, was highly variable between the strains and contained IS*1398* and/or various genes depending on the strain. Notably, only one copy of IS*1398* was present in the IS*1398*‐positive genomes, and an IS*1398* element was associated with a 5‐bp target site duplication, indicating a simple insertion of this IS element into this genomic locus. In this region, the *cif*_Yp_ gene was also present as previously reported (strain numbers 5, 9, and 14 in Figure 2; see Figure 1 for their phylogenetic positions and Tables S1 and S2 for strain information). Importantly, in the *ypm*‐positive European clade strain (strain YER_AA2585AA; strain number 16 in Figure 2; see Figure 1 and Table S2 for its phylogenetic position and strain information, respectively), the *ypmA* gene was located at the same position as in the *ypm*‐positive Asian and transitional clade strains (strain numbers 1, 2, 4, 6, and 7 in Figure 2). These genomic and phylogenetic features suggest that the *ypmA* gene was acquired by the Ypt lineage at a very early stage of its evolution, which was followed by a single amino acid substitution in the *ypmA* gene in a *ypmC*‐positive transitional clade lineage and the deletion of the *ypmA* gene in most transitional and European clade strains/lineages, although we can not exclude a possibility that *ypmA* was acquired later by the *ypmA*‐positive European clade strain.

## 4 Conclusion

We determined the WGSs of 35 Japanese Ypt strains, including 11 strains from KD patients. By adding these genomes to publicly available Ypt genomes, we constructed a genome set representing the near‐global population of Ypt and analyzed the phylogenetic positions of the KD‐associated strains in the determined near‐global population structure. All the sequenced Japanese strains, including the KD‐associated strains, belonged to the Asian clade, which appeared to be the ancestral clade of Ypt, but the KD‐associated strains belonged to multiple lineages in this clade, not any specific lineages. Strains from patients with FESLF, a KD‐related disease, also belonged to the Asian clade. Moreover, no KD strain‐specific genes were identified in pan‐GWAS analyses. Notably, however, the *ypm* gene encoding the YPM superantigen showed a distribution pattern highly biased to the Asian clade. This distribution pattern may be linked to the high incidence of KD in Japan and raises the possibility that Asian clade strains may have greater potential to trigger KD, although further studies on the roles of YPM and other environmental factors as well as host factors in the onset of KD are needed.

## Disclosure

The authors have nothing to report.

## Conflicts of Interest

The authors declare no conflicts of interest.

## Supporting information

Table S1 List of strains sequenced in this study.

Table S2 List of the genomes obtained from the NCBI database.

Table S3 List of major virulence factors analyzed in this study.

## Data Availability

The sequence data that support the findings of this study are openly available in GenBank/EMBL/DDBJ (https://www.ddbj.nig.ac.jp) under the BioProject accession number PRJDB17094. The sequence accession numbers of each strain are provided in Table S1.
