# Supplementary material for: Genome Analysis of Japanese Yersinia pseudotuberculosis Strains Isolated From Kawasaki Disease Patients and Other Sources and Their Phylogenetic Positions in the Global Y. pseudotuberculosis Population
Source: Microbiol Immunol. 2025 Jan 9;69(3):182–90. doi: 10.1111/1348-0421.13199 (PMC11873759; doi:10.1111/1348-0421.13199)
Supplement: Supplementary file 1 — Table S1 List of strains sequenced in this study. [file MIM-69-182-s002.pdf]

Table S1. List of strains sequenced in this study.

Strain name	Isolation source	Isolation date	Assembly Level	Genome size (bp)	Number of scaffolds/contigs	Length of chromosome (bp)	Length of plasmid (bp)	Accession number		
								**Illumina reads**	**ONT reads**	**Assembled sequence**
164	Human (enterocolitis)	2018	Draft	4,795,619	62	na	na	DRR527870	na	
1169	Human (enterocolitis)	2018	Draft	4,654,216	62	na	na	DRR527871	na	
Aichi	Human (enterocolitis)	2019	Draft	4,805,047	65	na	na	DRR527890	na	
Hyogo	Human (enterocolitis)	Unknown	Draft	4,800,152	60	na	na	DRR527889	na	
kawasaki_2	Human (enterocolitis)	2012	Draft	4,849,577	72	na	na	DRR527886	na	
KD1	Human (Kawasaki disease)	2015	Finished	4,705,119	1	4,705,119	na	DRR527864	DRR527918	AP031360
KD10	Human (Kawasaki disease)	Unknown	Draft	4,925,422	103	na	na	DRR527895	na	
KD11	Human (Kawasaki disease)	Unknown	Draft	4,871,849	73	na	na	DRR527896	na	
KD2	Human (Kawasaki disease)	2015	Finished	4,897,470	2	4,822,514	74,956	DRR527865	DRR527919	chromosome; AP031361<br>plasmid; AP031362
KD3	Human (Kawasaki disease)	2012	Finished	5,008,018	1	5,008,018	na	DRR527866	DRR527920	AP031363
KD4	Human (Kawasaki disease)	2012	Finished	4,911,601	2	4,836,613	74,988	DRR527867	DRR527921	chromosome; AP031364<br>plasmid; AP031365
KD5	Human (Kawasaki disease)	2011	Finished	4,911,284	2	4,836,274	75,010	DRR527868	DRR527922	chromosome; AP031366<br>plasmid; AP031367
KD6	Human (Kawasaki disease)	Unknown	Draft	4,867,454	84	na	na	DRR527869	na	
KD7	Human (Kawasaki disease)	2012	Draft	5,010,672	85	na	na	DRR527885	na	
KD8	Human (Kawasaki disease)	2015	Draft	4,926,305	75	na	na	DRR527887	na	
KD9	Human (Kawasaki disease)	2015	Draft	4,918,568	81	na	na	DRR527888	na	
4750	Human (enterocolitis)	2016	Draft	4,820,931	62	na	na	DRR527897	na	
okayama_1	Human (enterocolitis)	1981	Draft	4,946,585	107	na	na	DRR527872	na	
okayama_11477	Mouse	1993	Draft	4,858,929	85	na	na	DRR527881	na	
okayama_10811	Freshwater	1992	Draft	4,956,255	91	na	na	DRR527882	na	
okayama_10812	Freshwater	1992	Draft	4,794,745	72	na	na	DRR527883	na	
okayama_10403	Raccoon dog	1992	Draft	4,934,503	75	na	na	DRR527884	na	
okayama_2	Human (enterocolitis)	1999	Draft	4,855,835	83	na	na	DRR527873	na	
okayama_116	Cattle feces	2003	Draft	4,957,996	93	na	na	DRR527874	na	
okayama_3	dog	1999	Finished	4,811,060	1	4,811,060	na	DRR527875	DRR527923	AP031368
okayama_31	Dog	2000	Draft	4,679,907	82	na	na	DRR527876	na	
okayama_40	Cattle feces	2000	Draft	4,874,567	68	na	na	DRR527877	na	
okayama_9620	Raccoon dog	1991	Draft	4,722,385	89	na	na	DRR527878	na	
okayama_9524	Fox	1991	Finished	4,800,666	2	4,796,399	4,267	DRR527879	DRR527924	chromosome; AP031369<br>plasmid; AP031370
okayama_9172	Wild boar	1991	Draft	4,838,304	67	na	na	DRR527880	na	
6H56	Human (enterocolitis)	1994	Draft	4,701,862	87	na	na	DRR527891	na	
22H216	Human (enterocolitis)	2010	Draft	4,614,900	47	na	na	DRR527892	na	
6130	Human (enterocolitis)	Unknown	Draft	4,803,808	79	na	na	DRR527893	na	
32	Human (enterocolitis)	Unknown	Draft	4,734,422	69	na	na	DRR527863	na	
993	Human (enterocolitis)	2017	Draft	4,780,639	66	na	na	DRR527894	na	

na; not applicable
