# Supplementary material for: Genome Analysis of Japanese Yersinia pseudotuberculosis Strains Isolated From Kawasaki Disease Patients and Other Sources and Their Phylogenetic Positions in the Global Y. pseudotuberculosis Population
Source: Microbiol Immunol. 2025 Jan 9;69(3):182–90. doi: 10.1111/1348-0421.13199 (PMC11873759; doi:10.1111/1348-0421.13199)
Supplement: Supplementary file 2 — Table S2 List of the genomes obtained from the NCBI database. [file MIM-69-182-s001.pdf]

Table S2. List of strains whose genome sequences were obtained from the NCBI database.

Strain name	Region	Country	Isolation source	Accession number	Number of scaffolds/contigs	Genome size (bp)	Coverage	Assembly levels	Note
NZYP4678	Oceania	New Zealand	Feline	ERR1413977	109	4,713,180	115	Draft	
NZYP4679	Oceania	New Zealand	Feline	ERR1413978	107	4,750,238	109	Draft	
NZYP4680	Oceania	New Zealand	Human	ERR1413979	162	4,998,012	99	Draft	
NZYP4681	Oceania	New Zealand	Human	ERR1413980	93	4,724,036	111	Draft	
NZYP4682	Oceania	New Zealand	Human	ERR1413981	132	4,721,053	112	Draft	
NZYP4683	Oceania	New Zealand	Human	ERR1413982	58	4,405,873	104	Draft	
NZYP4684	Oceania	New Zealand	Human	ERR1413983	97	4,684,185	125	Draft	
NZYP4685	Oceania	New Zealand	Human	ERR1413984	48	4,378,017	115	Draft	
NZYP4686	Oceania	New Zealand	Human	ERR1413985	131	4,821,678	110	Draft	
NZYP4687	Oceania	New Zealand	Human	ERR1413986	123	4,758,494	114	Draft	
NZYP4688	Oceania	New Zealand	Human	ERR1413987	137	4,689,744	116	Draft	
NZYP4689	Oceania	New Zealand	Human	ERR1413988	102	4,936,500	101	Draft	
NZYP4691	Oceania	New Zealand	Human	ERR1413990	115	4,846,641	112	Draft	
NZYP4692	Oceania	New Zealand	Cervine	ERR1413991	45	4,413,249	119	Draft	
NZYP4693	Oceania	New Zealand	Cervine	ERR1413992	54	4,341,447	139	Draft	
NZYP4694	Oceania	New Zealand	Human	ERR1413993	99	4,693,237	116	Draft	
NZYP4695	Oceania	New Zealand	Human	ERR1413994	83	4,718,420	111	Draft	
NZYP4696	Oceania	New Zealand	Human	ERR1413995	77	4,717,467	105	Draft	
NZYP4697	Oceania	New Zealand	Human	ERR1413996	98	4,731,224	115	Draft	
NZYP4698	Oceania	New Zealand	Human	ERR1413997	110	4,724,481	112	Draft	
NZYP4699	Oceania	New Zealand	Human	ERR1413998	102	4,732,309	103	Draft	
NZYP4700	Oceania	New Zealand	Human	ERR1413999	93	4,861,117	96	Draft	
NZYP4701	Oceania	New Zealand	Human	ERR1414000	144	5,043,365	105	Draft	
NZYP4702	Oceania	New Zealand	Human	ERR1414001	93	4,608,987	122	Draft	
NZYP4703	Oceania	New Zealand	Human	ERR1414002	98	4,639,010	99	Draft	
NZYP4704	Oceania	New Zealand	Human	ERR1414003	120	4,789,395	94	Draft	
NZYP4705	Oceania	New Zealand	Human	ERR1414004	80	4,647,471	106	Draft	
NZYP4706	Oceania	New Zealand	Human	ERR1414005	128	4,710,865	84	Draft	
NZYP4709	Oceania	New Zealand	Human	ERR1414008	88	4,722,230	107	Draft	
NZYP4711	Oceania	New Zealand	Human	ERR1414010	85	4,724,108	113	Draft	
NZYP4717	Oceania	New Zealand	Human	ERR1414015	94	4,722,683	110	Draft	
NZYP4730	Oceania	New Zealand	Human	ERR1414028	97	4,722,962	110	Draft	
NZYP4757	Oceania	New Zealand	Human	ERR1414055	90	4,723,470	46	Draft	
NZYP4764	Oceania	New Zealand	Human	ERR1414062	82	4,658,166	100	Draft	
NZYP4766	Oceania	New Zealand	Human	ERR1414064	129	4,639,185	110	Draft	
NZYP4788	Oceania	New Zealand	Human	ERR1414086	104	4,721,867	112	Draft	
NZYP4792	Oceania	New Zealand	Human	ERR1414090	116	4,685,716	111	Draft	
NZYP4794	Oceania	New Zealand	Human	ERR1414092	132	4,769,566	107	Draft	
NZYP4795	Oceania	New Zealand	Human	ERR1414093	108	4,720,397	122	Draft	
NZYP4796	Oceania	New Zealand	Human	ERR1414094	100	4,723,672	117	Draft	
NZYP4798	Oceania	New Zealand	Human	ERR1414096	127	4,613,525	114	Draft	
NZYP8106	Oceania	New Zealand	Human	ERR1414098	147	4,578,466	148	Draft	
NZYP8108	Oceania	New Zealand	Human	ERR1414100	215	4,676,783	100	Draft	
Yptb-110	Europe w/o Finland	France	Unknown	ERR1448063	149	4,991,289	139	Draft	
S1	Finland	Finland	Bulk tank milk	ERR2713003	200	4,579,959	91	Draft	
S24	Finland	Finland	Cattle feces	ERR2713013	214	4,760,109	79	Draft	
S27	Finland	Finland	Cattle feces	ERR2713016	239	4,706,548	119	Draft	
2874/2003	Finland	Finland	Kitchen bench surface	ERR4425851	77	4,787,298	175	Draft	
H-1	Russia	Russia	Human (clinical)	ERR4425852	155	4,849,331	61	Draft	
YER_AA2543AA	Europe w/o Finland	Germany	Human (clinical)	ERR4425853	119	4,577,561	218	Draft	
YER AA2528AA	Europe w/o Finland	Germany	Duck	ERR4425854	122	4,665,607	119	Draft	
866/81	Finland	Finland	Human (clinical)	ERR4425855	133	4,813,337	147	Draft	
MW Taniguci	Japan	Japan	Well water	ERR4425856	160	4,791,409	132	Draft	
YER_AA2527AA	Europe w/o Finland	Belgium	Human (clinical)	ERR4425859	126	4,800,430	183	Draft	
YER_AA2515AA	Japan	Japan	Duck	ERR4425862	151	4,824,203	77	Draft	
YER_AA2530AA	Japan	Japan	Pig	ERR4425863	189	4,678,812	114	Draft	
PC94-72	Japan	Japan	Pig	ERR4425865	193	4,906,473	50	Draft	
R103-2	Asia w/o Japan	China	Rabbit	ERR4425866	169	4,734,717	51	Draft	
YER AA2532AA	Russia	Russia	Reindeer	ERR4425868	143	4,718,446	75	Draft	
2484/2006	Finland	Finland	Potato waste on storage room floor	ERR4425869	92	4,787,915	227	Draft	
No.93	Japan	Japan	Racoon dog	ERR4425870	231	4,679,209	30	Draft	
2886	Europe w/o Finland	Belgium	Human (clinical)	ERR4425871	191	4,732,235	34	Draft	
H938-36/89	Russia	Russia	Mus musculus	ERR4425877	181	4,875,457	39	Draft	
TP1039	Finland	Finland	Human (clinical)	ERR4425878	109	4,746,185	54	Draft	
504/72	Asia w/o Japan	China	Rabbit	ERR4425880	132	4,872,742	123	Draft	
79136	Russia	Russia	Rattus norvegicus	ERR4425881	218	4,914,492	55	Draft	
YER_AA2514AA	North America	Canada	Human (clinical)	ERR4425882	197	4,665,680	33	Draft	
YER_AA2552AA	Oceania	New Zealand	Goat	ERR4425883	130	4,711,664	127	Draft	
YER_AA2553AA	Europe w/o Finland	Italy	Hare	ERR4425884	108	4,561,610	116	Draft	

YER_AA2556AA	Europe w/o Finland	Germany	Hare	ERR4425885	141	4,694,950	44	Draft
YER_AA2538AA	Europe w/o Finland	Italy	Guinea pig	ERR4425886	142	4,726,695	183	Draft
YER_AA2572AA	Japan	Japan	Pig	ERR4425887	170	4,706,404	47	Draft
YER_AA2555AA	Europe w/o Finland	Italy	White peking duck	ERR4425888	195	4,694,535	33	Draft
YER_AA2546AA	Russia	Russia	Salmon	ERR4425890	144	4,731,692	118	Draft
YER_AA2562AA	Asia w/o Japan	South Korea	Human (clinical)	ERR4425892	154	4,726,323	93	Draft
H943-36/89	Europe w/o Finland	Germany	Hare	ERR4425893	163	4,598,931	33	Draft
Y.PT/8	Europe w/o Finland	Belgium	Human (clinical)	ERR4425895	147	4,627,323	44	Draft
No.21	Oceania	New Zealand	Cattle	ERR4425896	137	4,686,896	103	Draft
G2/77/2	Europe w/o Finland	Denmark	Bird	ERR4425897	134	4,756,373	100	Draft
2817/1998	Finland	Finland	Brown hare	ERR4425898	116	4,498,013	104	Draft
3876/2001	Finland	Finland	Hare	ERR4425900	191	4,555,760	32	Draft
3858/2000	Finland	Finland	Brown hare	ERR4425901	181	4,766,658	35	Draft
2161/13/2006	Finland	Finland	Dirst sample from storage room floor	ERR4425903	86	4,787,263	209	Draft
677/82	Finland	Finland	Human (clinical)	ERR4425904	210	4,993,035	52	Draft
Chigamatsu	Japan	Japan	Human (clinical)	ERR4425906	159	4,759,151	49	Draft
8011-3	Japan	Japan	Human (clinical)	ERR4425908	143	4,545,972	91	Draft
3822/2000	Finland	Finland	Brown hare	ERR4425909	172	4,628,127	35	Draft
5456/85	Finland	Finland	Human (clinical)	ERR4425910	195	4,996,257	58	Draft
Uematu289	Japan	Japan	Human (clinical)	ERR4425912	135	4,871,341	70	Draft
2814/1998	Finland	Finland	Brown hare	ERR4425914	120	4,675,347	89	Draft
GS951	Asia w/o Japan	China	Human (clinical)	ERR4425915	194	4,775,855	88	Draft
921/93	Europe w/o Finland	Sweden	Human (clinical)	ERR4425916	258	4,724,964	54	Draft
YER_AA2617AA	Europe w/o Finland	France	Unknown	ERR4425917	127	4,609,603	262	Draft
42/00	Europe w/o Finland	Sweden	Human (clinical)	ERR4425918	129	4,654,444	227	Draft
36/83	Finland	Finland	Human (clinical)	ERR4425919	142	4,817,195	64	Draft
YER_AA2603AA	Finland	Finland	Deer	ERR4425920	157	4,723,936	72	Draft
2884	Europe w/o Finland	Italy	Rabbit	ERR4425921	237	4,816,691	44	Draft
YER_AA2614AA	Europe w/o Finland	France	Unknown	ERR4425922	170	4,871,719	239	Draft
YER_AA2609AA	Europe w/o Finland	Sweden	Unknown	ERR4425923	105	4,678,115	257	Draft
Y.PT/7	Europe w/o Finland	France	Unknown	ERR4425924	56	4,297,717	228	Draft
YER_AA2619AA	Finland	Finland	Human	ERR4425925	126	4,702,268	107	Draft
YER_AA2513AA	North America	Canada	Human (clinical)	ERR4425926	160	4,828,779	57	Draft
YER_AA2511AA	Europe w/o Finland	Italy	Turkey	ERR4425927	125	4,635,106	83	Draft
YER_AA2512AA	South America	Brazil	Bovine	ERR4425933	73	4,360,720	50	Draft
YER_AA2531AA	Russia	Russia	Salmon	ERR4425934	142	4,735,044	369	Draft
T-469-1	Japan	Japan	Pig	ERR4425936	170	4,670,705	117	Draft
MW145-2	Japan	Japan	Mountain water	ERR4425937	160	4,857,021	146	Draft
YER_AA2533AA	Asia w/o Japan	China	Wild rat	ERR4425938	184	4,721,279	36	Draft
25418L	Europe w/o Finland	Denmark	Cary	ERR4425939	126	4,616,058	124	Draft
YER_AA2505AA	Finland	Finland	Human (clinical)	ERR4425942	116	4,778,755	153	Draft
YER_AA2509AA	Finland	Finland	Guinea pig	ERR4425943	128	4,680,280	102	Draft
YER_AA2519AA	Japan	Japan	Cat	ERR4425944	192	4,629,291	44	Draft
YER_AA2520AA	Japan	Japan	Racoon dog	ERR4425945	186	4,836,068	89	Draft
YER_AA2523AA	Russia	Russia	Human (clinical)	ERR4425947	151	4,866,049	76	Draft
YER_AA2524AA	Russia	Russia	Human (clinical)	ERR4425948	241	4,869,903	42	Draft
YER_AA2529AA	Japan	Japan	Dog	ERR4425949	165	4,686,596	159	Draft
YER_AA2535AA	Japan	Japan	Human (clinical)	ERR4425950	166	4,934,159	113	Draft
YER_AA2554AA	Europe w/o Finland	Italy	Rabbit	ERR4425957	140	4,802,178	67	Draft
H722-36/88	Europe w/o Finland	Belgium	Human (clinical)	ERR4425958	148	4,724,511	112	Draft
2497	Europe w/o Finland	Italy	Hare	ERR4425961	129	4,676,179	102	Draft
Gifu-liver	Japan	Japan	Monkey	ERR4425962	161	4,686,667	57	Draft
TE-93181	Japan	Japan	Racoon dog	ERR4425964	172	4,691,426	130	Draft
YER_AA2585AA	Europe w/o Finland	Denmark	Horse	ERR4425965	139	4,552,979	75	Draft
2809/1998	Finland	Finland	Hedgehog	ERR4425966	122	4,687,701	103	Draft
YER_AA2595AA	Finland	Finland	Brown hare	ERR4425967	135	4,645,802	111	Draft
7616/84	Finland	Finland	Human (clinical)	ERR4425968	116	4,675,344	112	Draft
598	Russia	Russia	Human (FESLF)*	SRR12808798	123	4,898,590	99	Draft
27802D-5	North America	USA	Unknown	SRR12825850	110	4,683,605	461	Draft
ATCC-29833	North America	USA	Unknown	SRR13386520	81	4,555,252	214	Draft
WAPHL-YPT-A00001	North America	USA	Unknown	SRR1922792	204	4,989,340	97	Draft
WAPHL-YPT-A00002	North America	USA	Unknown	SRR1922793	149	4,951,631	118	Draft
WAPHL-YPT-A00004	North America	USA	Unknown	SRR1922794	108	4,701,677	120	Draft
WAPHL-YPT-A00003	North America	USA	Unknown	SRR1922795	75	4,592,251	127	Draft
WAPHL-YPT-A00005	North America	USA	Unknown	SRR1922796	95	4,766,854	128	Draft
WAPHL-YPT-A00006	North America	USA	Unknown	SRR1922797	274	5,227,225	75	Draft
PNUSAY000005	North America	USA	Unknown	SRR6702975	88	4,768,778	89	Draft
PNUSAY000008	North America	USA	Unknown	SRR6702976	92	4,864,018	52	Draft
PNUSAY000007	North America	USA	Unknown	SRR6702977	85	4,806,628	55	Draft
Yptb-120	Europe w/o Finland	France	Unknown	ERR1448073	119	4,614,680	194	Draft

IP31758	Russia	Russia	Human (FESLF)*	CP000718.1, CP000719.1, CP000720.1	3	4,935,125	na	Finished	Chromosome; 4,723,306 bp, plasmids; 153,140 bp and 58,679 bp
IP32953	Europe w/o Finland	France	Human (clinical)	CP009710.1, CP009711.1, CP009712.1	3	4,840,898	na	Finished	Chromosome; 4,743,972 bp, plasmids; 68,525 bp and 26,933 bp
PA3606	Japan	Japan	Human	CP010067.1, CP010068.1, CP010069.1	3	4,830,299	na	Finished	Chromosome; 4,742,580 bp, plasmids; 74,956 bp and 12,763 bp
FDAARGOS_342	Europe w/o Finland	Sweden	Turkey	CP031780.1	1	4,905,554	na	Finished	Chromosome; 4905554 bp
FDAARGOS_583	Europe w/o Finland	France	Human (clinical)	CP033708.1, CP033709.1	2	4,652,218	na	Finished	Chromosome; 4,582,218 bp, plasmid; 69,970 bp
FDAARGOS_582	Europe w/o Finland	France	Human (clinical)	CP033710.1, CP033711.1	2	4,832,460	na	Finished	Chromosome; 4,762,645 bp, plasmid; 69,810 bp
FDAARGOS_581	Europe w/o Finland	France	Human (clinical)	CP033712.1, CP033713.1	2	5,096,964	na	Finished	Chromosome; 5026,929 bp, plasmid; 70,035 bp
FDAARGOS_580	Europe w/o Finland	France	Human (clinical)	CP033714.1, CP033715.1	2	4,981,010	na	Finished	Chromosome; 4,910,698 bp, plasmid; 70,312 bp
IP2666pIB1	North America	USA	Mouse	CP032566.1, CP032567.1	2	4,681,481	na	Finished	Chromosome; 4,614,856 bp, plasmid; 66,625 bp
NZYP4713	Oceania	New Zealand	Human (enterocolitis)	LT596221.1, LT596222.1	2	4,794,091	na	Finished	Chromosome; 4,724,276 bp, plasmid; 69,815 bp
NCTC3571	Europe w/o Finland	United Kingdom	Monkey	LR134306.1	1	4,938,759	na	Finished	Chromosome; 4938759 bp
NCTC10275	Europe w/o Finland	Sweden	Turkey	LR134373.1	1	4,610,200	na	Finished	Chromosome; 4610200 bp
FDAARGOS_665	North America	USA	Human (clinical)	CP044064.1	1	4,367,018	na	Finished	Chromosome; 4367018 bp
72344	Japan	Japan	Unknown	DRR287733	103	4,858,833	96	Draft	
1739912	Europe w/o Finland	United Kingdom	Human	SRR22407113	177	4,868,631	130	Draft	
882758	Europe w/o Finland	United Kingdom	Human	SRR22549894	190	4,699,201	104	Draft	
786555	Europe w/o Finland	United Kingdom	Human	SRR22549903	117	4,722,875	100	Draft	
882764	Europe w/o Finland	United Kingdom	Human	SRR22566358	159	4,776,678	64	Draft	
1781874	Europe w/o Finland	United Kingdom	Human	SRR22630791	178	4,784,162	121	Draft	
1785633	Europe w/o Finland	United Kingdom	Human	SRR22769403	191	4,800,268	86	Draft	
1826010	Europe w/o Finland	United Kingdom	Human	SRR23185572	194	4,548,745	103	Draft	
1125058	Europe w/o Finland	United Kingdom	Human	SRR24443270	195	4,627,461	129	Draft	
SP-1303	Russia	Russia	Mouse	SRR25411577	146	4,900,575	285	Draft	
1209605	Europe w/o Finland	United Kingdom	Human	SRR26188717	203	4,677,473	84	Draft	
1844935	Europe w/o Finland	United Kingdom	Human	SRR26188720	181	4,581,460	115	Draft	
1857711	Europe w/o Finland	United Kingdom	Human	SRR26188802	200	4,683,966	115	Draft	
1841175	Europe w/o Finland	United Kingdom	Human	SRR26188812	166	4,803,089	74	Draft	
1856230	Europe w/o Finland	United Kingdom	Human	SRR26188835	258	4,700,340	97	Draft	
1831201	Europe w/o Finland	United Kingdom	Human	SRR26188849	205	4,573,559	166	Draft	
1154533	Europe w/o Finland	United Kingdom	Human	SRR26188894	156	4,617,077	166	Draft	

*FESLF; Far East scarlet-like fever

na; not applicable
