# Supplementary material for: Genome Analysis of Japanese Yersinia pseudotuberculosis Strains Isolated From Kawasaki Disease Patients and Other Sources and Their Phylogenetic Positions in the Global Y. pseudotuberculosis Population
Source: Microbiol Immunol. 2025 Jan 9;69(3):182–90. doi: 10.1111/1348-0421.13199 (PMC11873759; doi:10.1111/1348-0421.13199)
Supplement: Supplementary file 3 — Table S3 List of major virulence factors analyzed in this study. [file MIM-69-182-s003.pdf]

Table S3. List of major virulence factors analyzed in this study

Virulence factor	Function	Sequence source: Strain	Accession No	Locus tag	Length (amino acid)	Gene location	Note	References
Inv	Invasin	IP32953	AJJ56932	BZ17_834	835	Chromosome		Johnson,S.L, et al. Genome Announc. 2015. Apr 30;3(2):e00148-15. doi: 10.1128
Ail	Attachment-invasion locus	IP32953	AJJ56628	BZ17_349	178	Chromosome		Johnson,S.L, et al. Genome Announc. 2015. Apr 30;3(2):e00148-15. doi: 10.1128
Cif_{Yp}	Cycle inhibiting factor	YPIII	A0A0H3B1Q8	YPK_1971	291	Chromosome		Jubelin G, et al. PLoS One. 2009;4(3):e4855. doi: 10.1371
YpmA	Superantigen	IP31758	ABS46381	ABS46381	151	Chromosome		Eppinger,M, et al. PLoS Genet. 2007 Aug;3(8):e142. doi: 10.1371
YpmC	Superantigen	YPT1	AAL02230	AF414083_1	151	Chromosome		Carnoy,C, et al. J. Bacteriol. 2002 Aug;184(16):4489-99. doi: 10.1128
Irp2	Iron-regulated protein	IP32953	AJJ54058	BZ17_914	3163	Chromosome	High Pathogenicity Island (HPI)	Johnson,S.L, et al. Genome Announc. 2015. Apr 30;3(2):e00148-15. doi: 10.1128
FyuA	Ferric yersiniabactin uptake receptor	IP32953	AJJ54301	BZ17_918	673	Chromosome	High Pathogenicity Island (HPI)	Johnson,S.L, et al. Genome Announc. 2015. Apr 30;3(2):e00148-15. doi: 10.1128
LcrF	Type III secretion system ATP synthase	IP32953	AJJ53131	BZ17_4267	439	Virulence plasmid		Johnson,S.L, et al. Genome Announc. 2015. Apr 30;3(2):e00148-15. doi: 10.1128
YopM	Type III secretion system effector	IP32953	AJJ53120	BZ17_4191	529	Virulence plasmid		Johnson,S.L, et al. Genome Announc. 2015. Apr 30;3(2):e00148-15. doi: 10.1128
